# Safety, Stability, and Therapeutic Efficacy of Long-Circulating TQ-Incorporated Liposomes: Implication in the Treatment of Lung Cancer

**DOI:** 10.3390/pharmaceutics14010153

**Published:** 2022-01-09

**Authors:** Arif Khan, Mohammed A. Alsahli, Mohammad A. Aljasir, Hamzah Maswadeh, Mugahid A. Mobark, Faizul Azam, Khaled S. Allemailem, Faris Alrumaihi, Fahad A. Alhumaydhi, Ameen S. S. Alwashmi, Ahmed A. Almatroudi, Mahdi H. Alsugoor, Masood A. Khan

**Affiliations:** 1 Department of Basic Health Sciences, College of Applied Medical Sciences, Qassim University, Buraydah 51452, Saudi Arabia; a_khan@qu.edu.sa; 2 Department of Medical Laboratories, College of Applied Medical Sciences, Qassim University, Buraydah 51452, Saudi Arabia; shly@qu.edu.sa (M.A.A.); mjasr@qu.edu.sa (M.A.A.); K.allemailem@qu.edu.sa (K.S.A.); f_alrumaihi@qu.edu.sa (F.A.); f.alhumaydhi@qu.edu.sa (F.A.A.); aswshmy@qu.edu.sa (A.S.S.A.); aamtrody@qu.edu.sa (A.A.A.); 3 Department of Pharmaceutics, College of Pharmacy, Qassim University, Buraydah 51452, Saudi Arabia; msodh@qu.edu.sa; 4 Department of Pharmacy Practice, College of Pharmacy, Qassim University, Buraydah 51452, Saudi Arabia; mu.mohammed@qu.edu.sa; 5 Department of Pathology, Faculty of Medicine, University of Kordofan, El-Obeid 157, Sudan; 6 Department of Pharmaceutical Chemistry and Pharmacognosy, Unaizah College of Pharmacy, Qassim University, Unaizah 51911, Saudi Arabia; f.azam@qu.edu.sa; 7 Department of Emergency Medical Services, Faculty of Health Sciences, Al Qunfudah, Umm Al-Qura University, Makkah 21912, Saudi Arabia; mhsugoor@uqu.edu.sa

**Keywords:** TQ-PEGylated liposomes, lung cancer cells, acute toxicity, molecular docking

## Abstract

Thymoquinone (TQ), which is one of the main bioactive constituents of *Nigella sativa* seeds, has demonstrated its potential against various cancer models. The poor solubility of TQ in aqueous solution limits its uses in clinical application. The present study aimed to develop a novel formulation of TQ to increase its bioavailability and therapeutic potential with minimal toxicity. Polyethylene glycol (PEG)-coated DSPC/cholesterol comprising TQ liposomes (PEG-Lip-TQ) were prepared and characterized on various aspects. A computational investigation using molecular docking was used to assess the possible binding interactions of TQ with 12 prospective anticancer drug targets. The in vitro anticancer activity was assessed in A549 and H460 lung cancer cells in a time- and dose-dependent manner, while the oral acute toxicity assay was evaluated in silico as well as in vivo in mice. TQ docked to the Hsp90 target had the lowest binding energy of −6.05 kcal/mol, whereas caspase 3 was recognized as the least likely target for TQ with a binding energy of −1.19 kcal/mol. The results showed 96% EE with 120 nm size, and −10.85 mv, ζ-potential of PEG-Lip-TQ, respectively. The cell cytotoxicity data demonstrated high sensitivity of PEG-Lip-TQ and a several fold decrease in the IC_50_ while comparing free TQ. The cell cycle analysis showed changes in the distribution of cells with doses. The in vivo data revealed an ~9-fold increase in the LD_50_ of PEG-Lip-TQ on free TQ as an estimated 775 and 89.5 mg/kg b.w, respectively. This study indicates that the pharmacological and efficacy profile of PEG-lip-TQ is superior to free TQ, which will pave the way for an exploration of the effect of TQ formulation in the treatment of lung cancer in clinical settings.

## 1. Introduction

According to Global Cancer Observatory, cancer is the second leading cause of mortality in developed as well as developing countries, as 9.5 million deaths associated with cancer were recorded worldwide [1]. The statistical data from GLOBOCAN 2021 suggested that breast and lung cancer are the most commonly diagnosed cancer among all types at 11.7% and 11.4%, respectively. However, the latter was reported as the leading cause of death with an estimated 1.8 million mortalities (18%) [1].

Several strategies have been growing to develop a novel anticancer drug with a high therapeutic index and minimal toxicity [2,3]. The use of secondary metabolites has shown immense development for the treatment of various types of diseases, including cancer, due to the claim of their no toxic effect. As most of the secondary metabolites are dietary constituents, these are also known as nutraceuticals [4,5]. The data from several studies suggested that 33% of cancer deaths could be saved by changing dietary habits with a high amount of natural foods [6]. Keeping this fact in consideration, drugs from natural sources have been used traditionally since ancient times in different parts of the world [7]. Furthermore, 50% of drugs that are available in the market are either derived from plants or chemically adapted [8,9]. Thymoquinone (TQ) is one of main bioactive constituents of black seeds (*Nigella sativa*), which has been shown to have many health-promoting benefits, including cancer prevention and treatment [10,11,12]. The use of black seeds and its oil has a long history in the treatment of several diseases as antidiabetic, antibacterial, antifungal, anticancer, etc. in South Asian, Arab, African, and Mediterranean countries [13,14]. As evident from various studies, TQ has been reported to be a remarkable therapeutic agent affecting multiple molecular pathways that regulate cellular proliferation, apoptosis, and metastasis [12,15,16]. However, irrespective of its wide therapeutic efficacy, the clinical application of TQ is limited due to its poor solubility in aqueous medium. Therefore, an appropriate formulation of TQ is necessary to explore the potential of this amazing agent against several diseases [17,18]. We recently reported the therapeutic potential of Lip-TQ against drug-sensitive and drug-resistant *A. baumannii* infections in the lungs of mice [19]. Earlier, we characterized the TQ-loaded liposomes and evaluated its therapeutic efficacy against the systemic infection of *Candida albicans* in diabetic mice [20]. Several studies also suggested the therapeutic potential and/or synergistic effect of TQ, while being incorporated in liposomes against a breast cancer system [21,22]. Nevertheless, the development of liposomes to target extra-RES (reticular endothelial system) is one of the major challenges as it should be taken minimally by RES. The coating of the liposomes with polyethylene glycol enhances the stability of the liposomes and protects it from recognition by RES. These long-circulating PEGylated liposomes are also called sterically stabilized or stealth liposomes. The optimal stability of the liposomal formulations can retain the entrapped drug in circulation for a long time, reach the target sites, and exert its cytotoxic activities in cancer cells [23,24,25].

However, it has been noticed that most of the secondary metabolites have not been as evaluated as much as other synthetic drugs at the toxicity level due to the misconception of ‘no toxicity’ of herbal medicines [26,27,28,29]. If any of the secondary metabolite has been found as a potential therapeutic agent, an evaluation of its LD_50_ and safety level of the doses is required. It has been found that in vivo acute and sub-acute oral toxicity assay is one of the most significant approached to determine the LD_50_ as well as the safety dose level [30,31,32]. Recently, in silico determination of the LD_50_ and safety dose level has also been given attention, which can help in dose selection and possible molecular targets of the drug [33,34]. Moreover, the use of computational and molecular modeling techniques has evolved in close parallel with experimentation. Homology modeling, density functional theory, quantitative structure–activity relationships, molecular docking, and molecular dynamics are some of the in silico approaches used to comprehend the molecular features of biological systems [35,36].

Our study was focused on the development and characterization of long-circulating TQ liposomes and their anticancer efficacy against the lung cancer system in vitro. We also evaluated in vivo acute oral toxicity of free and liposomal TQ to determine its LD_50_ and safety dose level. In addition, the in silico toxicity of free TQ was also estimated. Molecular docking was utilized to interpret the molecular interactions of TQ with lung cancer therapeutic targets.

## 2. Methods

### 2.1. Reagents

Distearoyl phosphatidylcholine (DSPC), 1,2-distearoyl-*sn*-glycero-3-phosphatiylethanol-amine-N-[methoxy(polyethyleneglycol)-2000] (DSPE-PEG_2000_), and Cholesterol (Chol) were procured from Sigma-Aldrich (St. Louis, MO, USA). The Cell Cytotoxicity Assay kits (ab112118), Benzo [a] Pyrene (BaP) were, purchased from Abcam (Cambridge, USA). Dulbecco’s Modified Eagle Medium (DMEM) and Fetal Bovine Serum (FBS) were purchased from Life Technologies, USA. A549 (ECACC 86012804), NCL-H358 (ECACC 95111733) were commercially purchased from ECACC (European Collection of Cell Cultures), Salisbury, UK.

### 2.2. Preparation of Thymoquinone Entrapped Liposomes

TQ encapsulated DSPC/Chol/mPEG-DSPE comprising long-circulating stealth liposomes were prepared as described in our previous studies with minor modifications [20,37]. Briefly, the DSPC: Chol (49:21) mmoles with mPEG-DSPE (5% of total phospholipids) and TQ (1% of total phospholipids) were prepared by the lipid film method. All the ingredients were mixed in a round bottom flask and then the solvents were evaporated to make the thin lipid film using a rotary evaporator in an N_2_ environment. The multilamellar vesicles (MLVs) were prepared by hydrating the lipid film with PBS followed by sonication to make ULVs using a probe sonicator. The suspension of ULVs was then extruded using a handheld extruder at ambient temperature sequentially from 400, 200, and 100 nm decreasing pore-sized polycarbonate membranes with 5–10 cycles for each size of the membrane. The unentrapped TQ was removed as the supernatant was discarded following centrifugation of TQ containing liposomes at 30,000 rpm for 30 min.

### 2.3. Molecular Docking Studies

We collected three-dimensional crystal structures of various anticancer targets from the Research Collaboratory for Structural Bioinformatics Protein Data Bank (RCSB PDB, http://www.rcsb.org/PDB, accessed on 20 September 2021). Table S1 lists all of the targets, their PDB IDs, and docking predictions for their binding energies. Biovia Discovery Studio Visualizer 2020 was used to remove co-crystallized water molecules, ligands, and cofactors from each protein in turn. After adding Gasteiger charges in MGLTools 1.5.6, the pdbqt file was saved. In MGL Tools 1.5.6, we defined the torsion tree and rotatable bonds in the TQ’s three-dimensional chemical structure, which we then translated to pdbqt format from the sdf format originally received from PubChem. Using Autogrid 4, we created grid maps of the binding sites in each target based on the native co-crystallized ligands. For 10 separate docking runs, AutoDock 4.2 was utilized with the Lamarckian genetic algorithm technique and the default docking protocol [38]. To identify the optimum postures of TQ in each target, we examined the binding energy (Δ*G*_binding_, kcal/mol) and non-bond interaction profiles of the top models of the ligand. Chimera 1.15, Pymol 1.7.4, and Biovia Discovery Studio Visualizer 2020 were used to study the molecular interactions as reported earlier [39,40].

### 2.4. Characterization of Liposomes

#### 2.4.1. The Size, Zeta (*ζ*) Potential (mV), and Poly Dispersity Index (PDI) and Entrapment Efficiency (EE) of TQ Containing and Empty Liposomes

The entrapment efficiency (EE) of TQ was determined by taking the absorbance at 330 nm using a standard plot of TQ [41]. The concentration of TQ in the liposomes was estimated after the disruption of liposomes with 0.5% Triton X-100. The percentage EE of TQ was determined using the following formula:

$$\%\;\mathrm{Entrapment}\;\mathrm{Efficiency}\;\left(\mathrm{EE}\right)\mathrm{of}\;\mathrm{the}\;\mathrm{drug}=\frac{\mathrm{Liposome}\;\mathrm{entrapped}\;\mathrm{drug}}{\mathrm{Total}\;\mathrm{drug}}\times 100$$

The mean particle sizes, zeta potentials, and PDI of prepared liposomes were determined by dynamic light scattering (DLS) using the Zetasizer Nano system (Malvern Instruments, Malvern, Worcestshire, UK).

#### 2.4.2. In Vitro Stability of Liposomes and Release Kinetics of TQ

The stability of TQ-stealth liposomes was determined by an in vitro drug release assay at 37 °C as described earlier [19]. Briefly, 1 mL of TQ containing PEGylated liposomes were taken in dialysis bags (MWCO 3.5 kDA), and dialyzed for the next 24 h against 20 mL of PBS with constant slow stirring. The sample (1 mL) was collected at various time points of 1, 2, 4, 8, 12, 18, and 24 h and replaced with 1 mL of PBS. The leakage of TQ was estimated by the following formula after determining the concentration of TQ by taking the absorbance at 330 nm in a UV-visible spectrophotometer.

The release kinetics of TQ from the PEGylated liposomes was assessed by incubating the TQ containing liposomes in 90% bovine serum. The reaction mixtures were then incubated for 1, 2, 4, 8, 12, and 24 h separately as described earlier [19]. The mixture was then centrifuged immediately after the respective incubation period at 25,000 rpm for 20 min. The leakage and release of TQ in the PBS and serum, respectively, were estimated by taking the absorbance at 330 nm in a UV-visible spectrophotometer by applying the following formula:

$$\mathrm{TQ}\;\mathrm{release}\;(\%)=\frac{\mathrm{CnV}+\sum_{i=0}^{n}\mathrm{CiVi}}{w}\times 100\%$$

where Cn is the concentration of TQ in the solution at the ‘n’ sampling point and Ci is the concentration of TQ in the solution at the ‘I’ sampling point. V is the total solution (20 mL) and Vi is the withdrawn volume every time (1 mL).

### 2.5. Cell Cytotoxicity Assay

The percentage of viability at varying concentrations of TQ was assessed to determine the IC_50_ of free as well as liposomal TQ in the A549 and H460 lung cancer cell lines. Briefly, the cells were seeded following 70–80% exponential confluency into 96-well cell culture plates (10,000 cells/well) for 24 h. The cells were then treated with varying concentrations of TQ as from 0.1–100 μM and incubated at 37 °C in a 5% CO_2_ atmosphere for 24, 48, and 72 h. As per the manufacturer’s instructions, the cell cytotoxicity reagent (20 μL) was added after the treatment for the respective period in each well, and then incubated at 37 °C followed by the measurements at 590 nm in a microplate reader. The viability of the cells was measured using the following formula:

$$\%\;\mathrm{Cell}\;\mathrm{Viability}=100\times \frac{\left(A_{\mathrm{sample}}-{\;A}_{0}\right)}{\left(A_{\mathrm{Ctrl}}-{\;A}_{0}\right)}$$

A_sample_ is the absorbance of FSE-treated cells.

A_ctrl_ is the absorbance of untreated cells.

A_0_ is the absorbance of the background of non-cell control (only media).

### 2.6. Cell Cycle Distribution Analysis

The IC_10_, IC_25_, and IC_35_ of PEG-Lip-TQ were selected to analyze the effect of liposomal formulations of TQ on the cell cycle distribution in A549 and H460 lung cancer cell lines by flow cytometry. The doses were measured as IC_10_ (3μM), IC_25_ (5 μM), and IC_35_ (7.2 μM) in A549, while 5, 6.5, and 10 μM, respectively, in H460 cells. Briefly, 2.5 × 10^5^ cells/well were seeded in the 6-well plates for 24 h, and then treated with selected doses of PEG-Lip-TQ. The cells were harvested, washed with sample buffer, and followed by fixation in 70% ice-cold ethanol in sample buffer overnight. The cells were suspended in the sample buffer containing 50 µg/mL propidium iodide (PI) and 100 µg/mL Ribonuclease A (RNAse A) followed by incubation at 37 °C for 30 min. The samples were acquired by MACSQuant Analyzer 10, and the cytometry results were analyzed using FlowJo software v10.8.1.

### 2.7. Toxicity Study of TQ

#### 2.7.1. In Silico Oral Toxicity Assay

The ProTox-II web server and T.E.S.T. program, Version 5.0.1, were used to analyze the toxicity and lethal dose (LD_50_) of thymoquinone [42,43]. Smiles ID of the chemical structure served as input in both programs. ProTox-II employs machine learning techniques to predict various toxicities, whereas the T.E.S.T. tool is based on advanced quantitative structure–activity relationship models to estimate the toxicity of natural or synthetic compounds, and their applicability is widely accepted [44,45].

#### 2.7.2. In Vivo Oral Acute Toxicity Assay

Female Swiss mice (8–10 weeks) were obtained from the animal house facility of the King Saud University, Riyadh, Saudi Arabia. The experiments involving the animals were carried out after the approval of the animal ethics committee of the College of Applied Medical Sciences, Qassim University, following the guidelines of the University of London Animal Welfare Society, Wheathampstead, England. All 57 mice were fasted overnight before the treatment and the food was provided approximately one hour after the treatment. The acute oral toxicity of TQ was investigated in the mice as the methods described by Lorke 1983 in two phases as shown in Table 1 [31]. In the first phase, three mice in each group were administered TQ through oral gavage as 0, 10, 100, and 1000 mg/kg b.w. in 200 μL of PBS, and observed for the next 14 days. The doses of the 2nd phase were prepared as stated in Table 1, according to the observational data of phase 1, suggested by Lorke 1983 [31]. The doses of PEG-Lip-TQ more than 1000 mg/kg b.w were administered in two to three times with the 4-h interval in 200 μL of PBS each time. The animals were exposed to 200 μL PBS in TQ 0, while 200 μL of empty liposomes were given to PEG-Lip-TQ 0. All the animals included in the study in both phases were observed regularly from the day of the exposure for two weeks. At the end of 14 days, all surviving mice were sacrificed, and then further studies were conducted to assess the toxicity of free TQ and PEG-Lip-TQ.

The LD_50_ was calculated by the formula:

$${\mathrm{LD}}_{50}=\sqrt{D_{0}\times D_{100}}$$

D_0_ = Highest dose that gave no mortality.

D_100_ = Lowest dose that produced mortality.

#### 2.7.3. Assessment of Relative Organ Weight

All the mice were sacrificed after the completion of the 14-day experimental period. The vital organs (lung, liver, kidney, heart, and spleen) were excised, and relative organ weight (ROW) was measured using the formula as follows:

$$\mathrm{ROW}=\frac{\mathrm{Organ}\;\mathrm{weight}}{\mathrm{Body}\;\mathrm{weight}}\times 100$$

#### 2.7.4. Hematological Analyses

The mice blood was evaluated for its total leucocyte count (TLC) and differential leucocyte count (DLC).

### 2.8. Statistical Analysis

The mean values and standard errors for all samples were calculated for different treated groups. The significant difference between the groups was measured by the one-way and two-way ANOVA, Tukey’s multiple comparison tests using Prism 9. *p*-value < 0.05 was considered statistically significant.

## 3. Results

### 3.1. Identification of Predicted Anticancer Targets of TQ by Molecular Docking Studies

**Figure 1:**
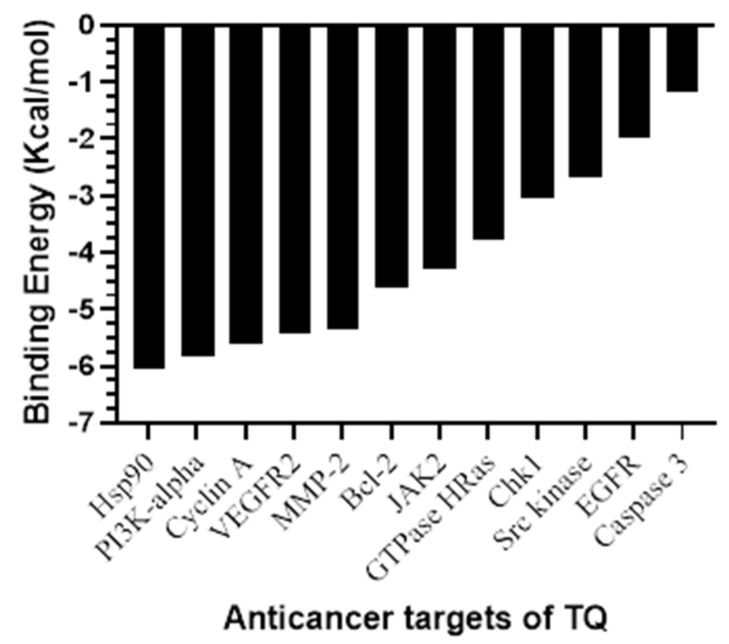
A bar plot of molecular docking─predicted binding energy (in kcal/mol) of TQ against potential anticancer drug targets.

**Figure 2:**
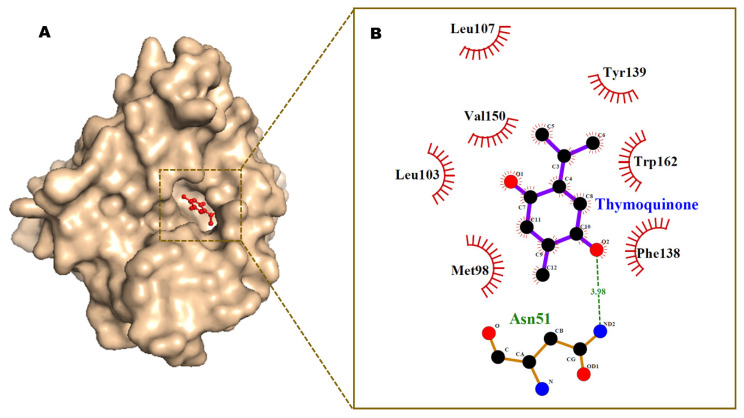
Minimum energy conformation of the docked TQ in the inhibitor binding cavity of Hsp90 protein shown as surface (**A**). Ligplot diagram (**B**) showing intermolecular interactions. Hydrogen bond interaction is shown as a broken green line.

**Figure 3:**
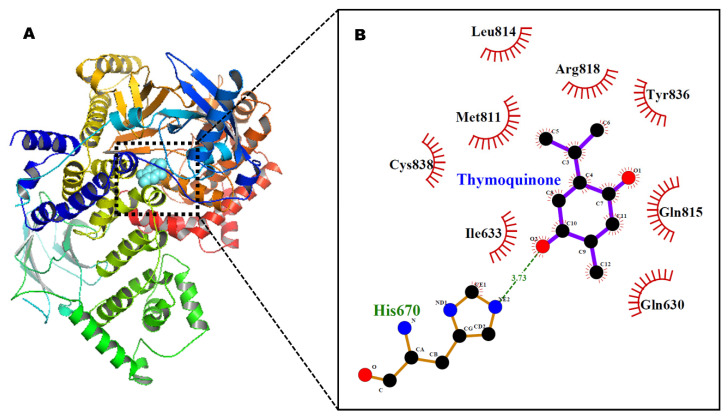
Docked TQ in the binding pocket of phosphatidylinositol-3 kinase alpha, shown in ribbon style (**A**). Ligplot diagram (**B**) showing intermolecular interactions. Hydrogen bond interaction is shown as a broken green line.

**Figure 4:**
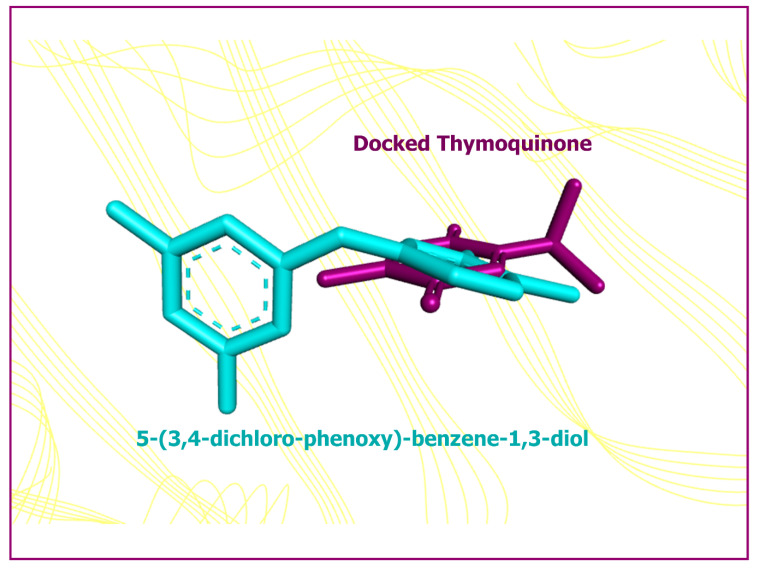
Superimposition of the minimum energy conformation of the docked TQ (shown in purple color) and the native co-crystallized inhibitor, 5-(3,4-dichloro-phenoxy)-benzene-1,3-diol (shown in cyan color), displaying a root-mean square deviation of 3.23 Å. The Hsp90 protein is shown as line ribbon in a faded yellow color.

The results of AutoDock 4.2 predicted affinity of TQ against 12 potential anticancer drug targets are presented in Figure 1. The binding energy ranged from −6.05 kcal/mol for Hsp90 protein to −1.19 kcal/mol against caspase-3. Appreciable affinity was observed, with PI3K-alpha, cyclin A, VEGFR2, and MMP-2 targets showing binding energy in the range of −5.32 to −5.82 kcal/mol (Table S1). The intermolecular interactions of docked TQ with heat shock protein-90 (Hsp90) and phosphatidylinositol-3 kinase (PI3K) alpha are portrayed in Figure 2 and Figure 3, respectively. In Hsp90 protein, docked TQ is surrounded by amino acid residues, such as Asn51, Met98, Leu103, Leu107, Phe138, Tyr139, Val150, and Trp162. The hydrophobic cleft is formed by all of these residues except Asn51, which acts as a donor of the hydrogen bond for TQ. The binding pocket of PI3K-alpha is constituted by Gln630, Ile633, His670, Met811, Leu814, Gln815, Arg818, Tyr836, and Cys838. Among these, His670 contributed a polar hydrogen bond with the TQ while the rest of the amino acid residues participated in hydrophobic interactions. In addition, docked TQ aligned itself within the Hsp90 binding pocket corresponding to the bound inhibitor, 5-(3,4-dichloro-phenoxy)-benzene-1,3-diol (Figure 4).

### 3.2. Characterization of Liposomes

#### 3.2.1. Size, PDI, ζ Potential, and EE

**Figure 5:**
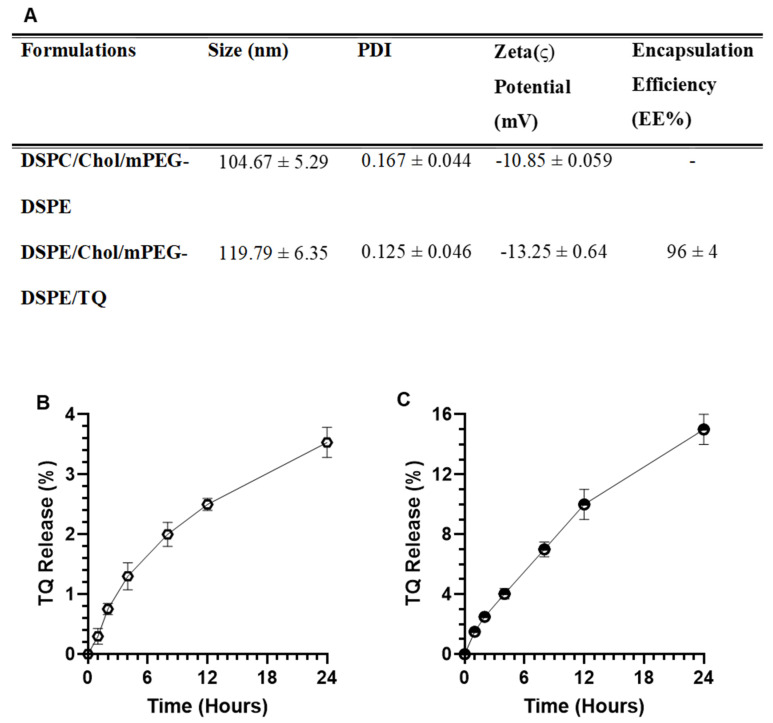
Characterization and in vitro stability and release kinetics of PEG-Lip-TQ. (**A**) size, PDI, Zeta Potential, and Entrapment Efficiency. (**B**) Stability of PEG-Lip-TQ in PBS. (**C**) Release kinetics of TQ from the liposomes into the serum. The values are expressed as mean ± SE of three independent experiments.

As shown in Figure 5, the DLS data showed that the mean particle size of DSPC/Chol containing PEGylated empty liposomes is 104 nm, while TQ entrapped liposomes were measured to be ~120 nm with <0.2 PDI homogeneity in both types of liposomes. The PDI and zeta potential of empty liposomes were measured as 0.167 and −10.85 mv, respectively. However, the PDI, zeta potential, and entrapment efficiency TQ-Lip were recorded as 0.125, −13.25 mv, and 96%, correspondingly (Figure 5A).

#### 3.2.2. In Vitro Stability of Liposomes and Release Kinetics of TQ

As depicted in Figure 5B, the data showed the stability of Lip-TQ as only 3.53% leakage of TQ at 37 °C in the PBS. The results from the release kinetics of TQ in the serum revealed that 1.5%, 2.5%, 4.03%, 7.0%, 10%, and 15% TQ was released after 1, 2, 4,8, 12, and 24 h, respectively (Figure 5C).

### 3.3. Effect of TQ on Cellular Proliferation and IC_50_ at Varying Doses of Free TQ and Lip-TQ in A549 and H460 Lung Cancer Cell Lines

**Figure 6:**
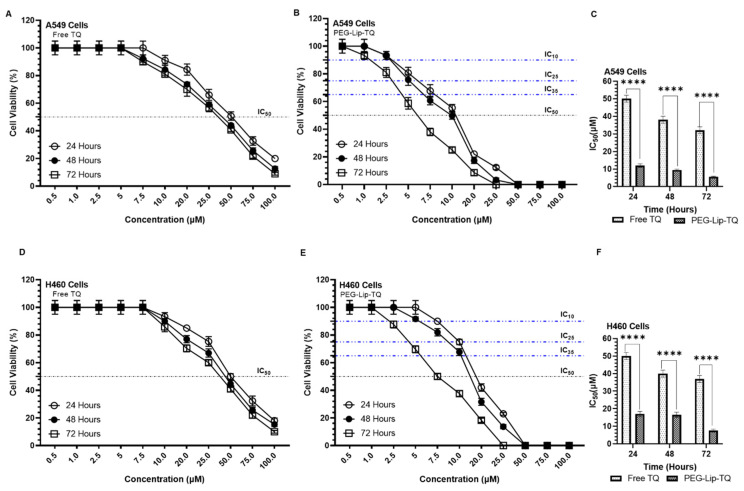
Effect of TQ at varying concentrations on cellular proliferation by cell cytotoxicity assay at 24, 48, and 72 h in A549 and H460 lung cancer cell lines. (**A**) Free TQ, (**B**) PEG-Lip-TQ, (**C**) IC_50_ of Free TQ, and PEG-Lip-TQ in A549 lung cancer cells, (**D**) Free TQ, (**E**) PEG-Lip-TQ, (**F**) IC_50_ of Free TQ, and PEG-Lip-TQ in H460 lung cancer cells. The values are expressed as mean ± SE of three independent experiments. **** Significant difference between the treated groups, *p*-value < 0.0001.

The results from the cell cytotoxicity assays revealed high sensitivity of TQ by PEG-Lip-TQ in comparison to free TQ (Figure 6). The data showed that the IC_50_ of free TQ was calculated as 50, 38, and 32 μM after 24, 48, and 72 h, respectively, in A549 lung cancer cells. However, the LD_50_ of Lip-TQ was found to be 12, 9.5, and 5.56 μM correspondingly, showing a significant difference at *p* < 0.0001 (Figure 6A–C). As depicted in Figure 6D–F, PEG-Lip-TQ was found to be significantly very effective as 17, 16, and 7.5 μM, when IC_50_ were measured after 24, 48, and 72 h, respectively, while 50, 40, and 37 μM were determined, correspondingly by free TQ. The results clearly indicated the cytotoxic effect of PEG-Lip-TQ as well as its stability as less than 40% and 55% μM concentrations were required to reach to the IC_50_ in A549 and H460 cells, respectively (Figure 6).

### 3.4. Effect of PEG-Lip-TQ on Cell Cycle Distribution at IC_10_, IC_25_, and IC_35_ in A549 and H460 Lung Cancer Cell Lines

**Figure 7:**
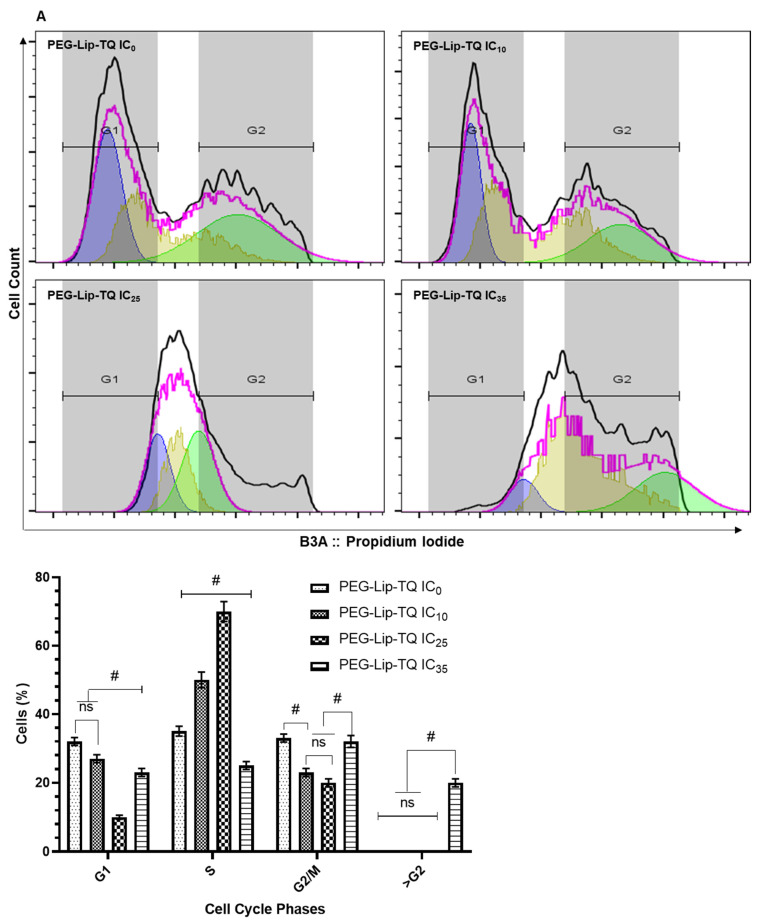

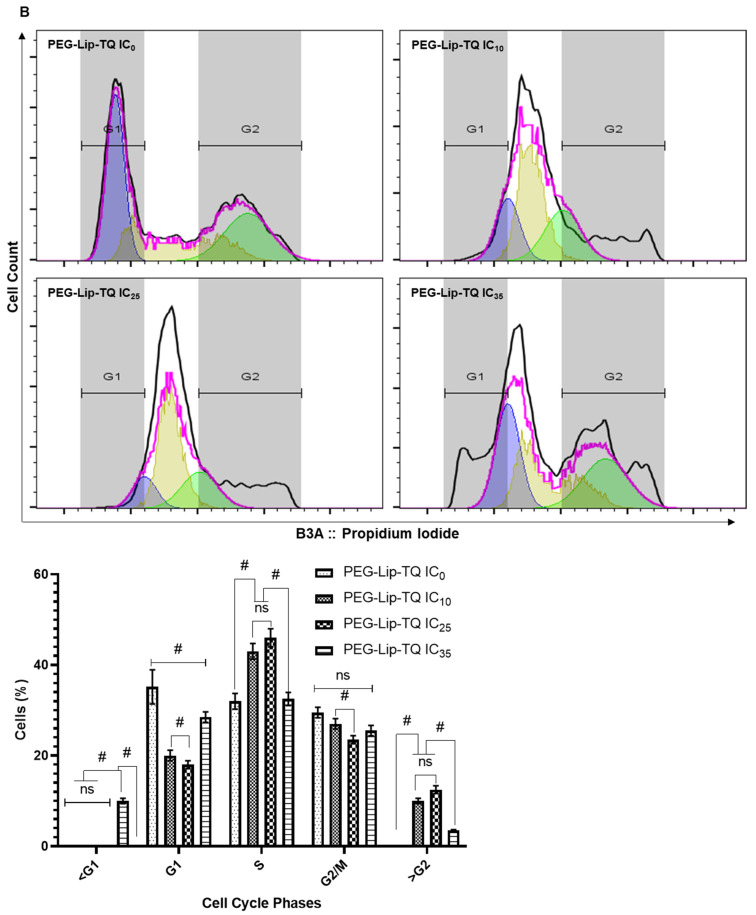
Effect of PEG-Lip-TQ cell cycle distribution by flow cytometry in lung cancer cells. (**A**) A549 cells, (**B**) H460 cells. The doses of PEG-Lip-TQ were selected as IC_10_ and IC_25_ and IC_35_ as in the materials and methods. The values are expressed as mean ± SE of three independent experiments. ^NS^ No Significance within the groups, ^#^ Significant difference between the treated groups.

The cell cycle analysis results showed the continuous changes in the distribution of cells treated with varying concentrations of PEG-Lip-TQ in both of the cells (Figure 7). As depicted in Figure 7A, 50% and 70% cells were arrested in the S-phase when treated with IC_10_ and IC_25_ 15% and 35% cells. However, the 20% cells were measured in >G2 phase in the cells treated with IC_35_. Similarly, a different cell cycle distribution was observed in the H460 cells treated with the formulation. As shown in Figure 7B, 43% of cells were arrested in S-phase and 10% were estimated in >G2 phases when treated with PEG-Lip-TQ IC_10_. The same pattern was noticed in the PEG-Lip-TQ IC_25_-treated cells as 46% of cells in S-phase and 12.55 in >G2 phase. Interesting, 10% cells were analyzed in <G1, and 3.5% in >G2 while treated with IC_35_ (Figure 7B).

### 3.5. In Vivo Oral Toxicity Study of TQ

#### 3.5.1. In Situ Toxicity Assay

Several toxicity parameters, such as hepatotoxicity, carcinogenicity, cytotoxicity, immunotoxicity, mutagenicity, and toxicities associated with stress response and nuclear receptor signaling pathways, were estimated by ProTox-II, which exploits fragment similarity-based cluster cross-validation and machine learning approaches. Thymoquinone was found to be inactive in all of the tested toxicities except mitochondrial membrane potential, a stress response pathway, in which it was found to be active, having a probability score of 0.58 (Table 2. The predicted LD_50_ of thymoquinone was noted as 2400 mg/kg, ranking V in toxicity class, according to the globally harmonized classification system in which class I represents the most toxic substance whereas class VI is regarded as a non-toxic entity [30,32].

Furthermore, the acute toxicity of thymoquinone was also determined using the T.E.S.T. program, which predicts the LD_50_ (oral lethal dosage for 50% of test rats) and mutagenicity of compounds. The obtained data are summarized in Table 3. The algorithm implemented in the T.E.S.T. program uses three distinct methods for predictions, which includes hierarchical clustering, consensus, and the nearest neighbor method. According to the consensus, hierarchical clustering, and nearest neighbor algorithms, the projected oral rat LD_50_ was 816.9, 1004.5, and 664.4 mg/kg, respectively, and negative mutagenicity was anticipated for the TQ.

#### 3.5.2. In Vivo Oral Toxicity Assay in Swiss Albino Mice

##### General Appearance, Behavioral Observations and LD_50_

**Figure 8:**
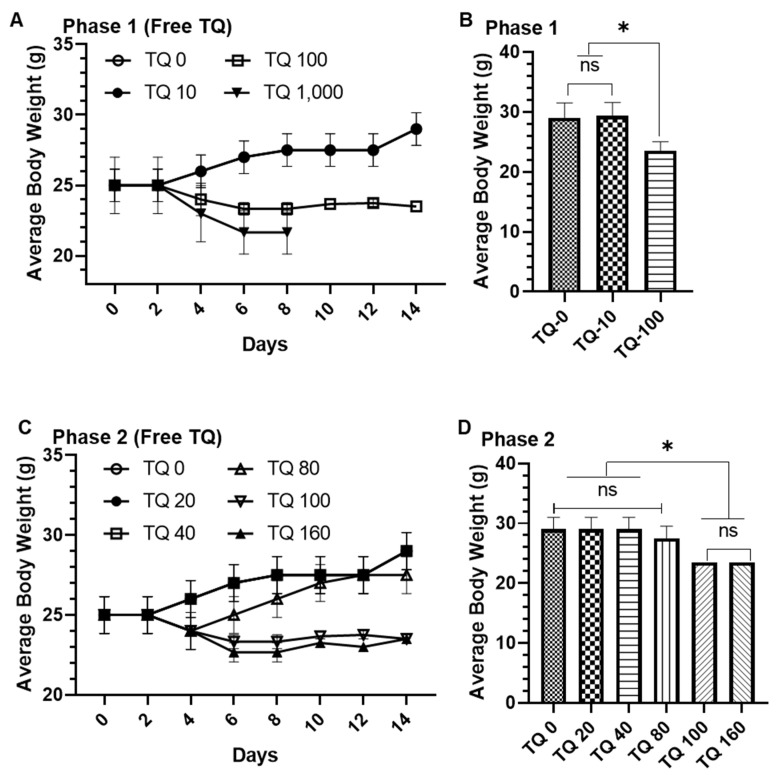

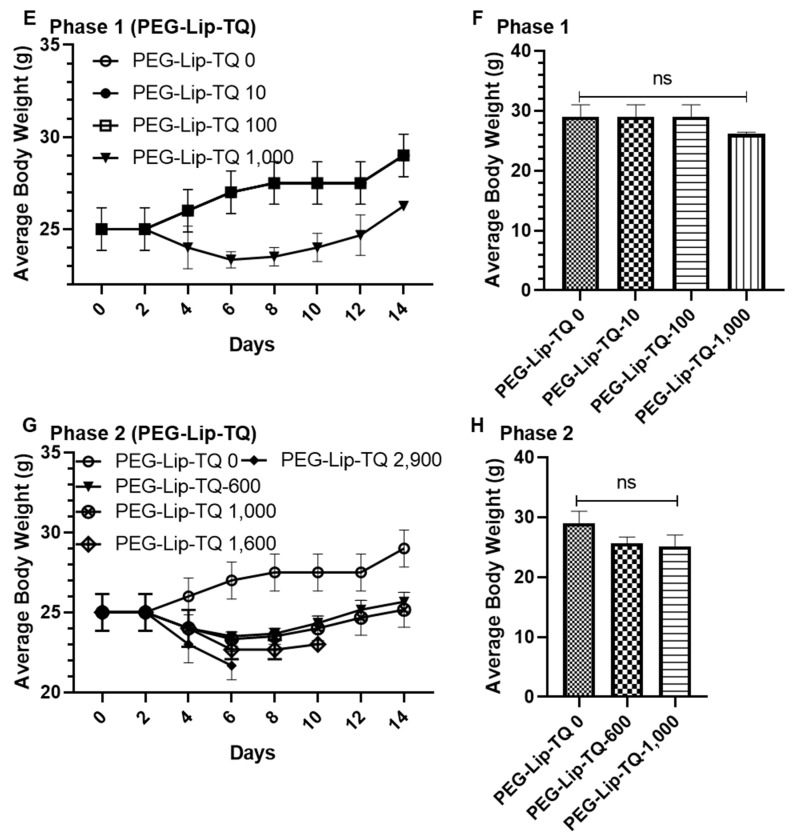

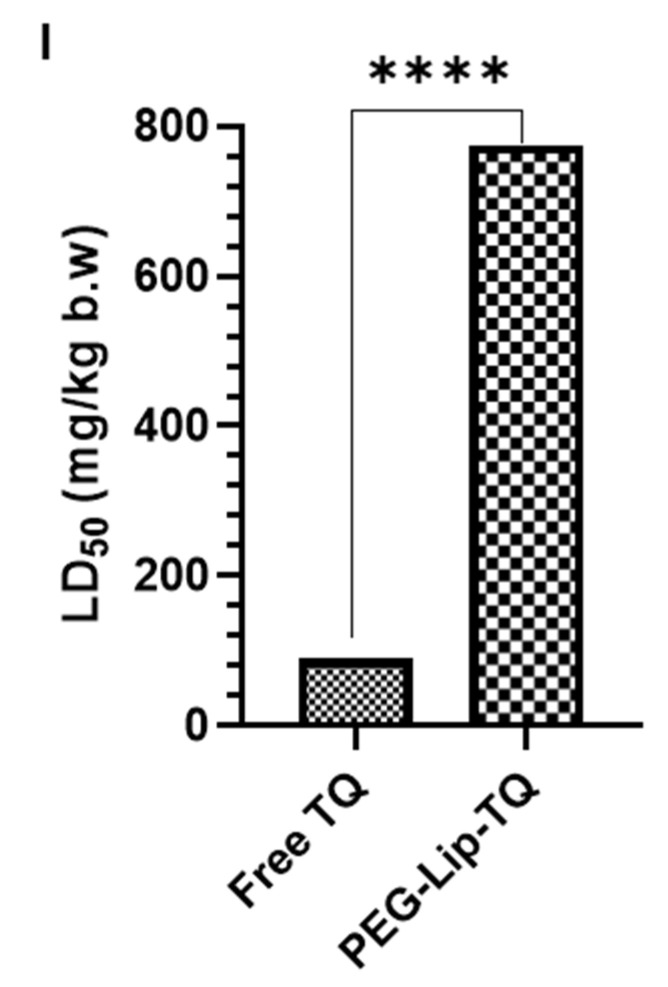
Effect of free TQ and PEG-Lip-TQ on body weight and LD_50_ (**A**) Average body weight by free TQ during the experiment period in phase 1, (**B**) Average body weight of surviving animals by free TQ after 14 days in phase 1, (**C**) Average body weight by free TQ during the experiment period in phase 2, (**D**) Average body weight of surviving animals by free TQ after 14 days in phase 2, (**E**) Average body weight by PEG-Lip-TQ during the experiment period in phase 1, (**F**) Average body weight of surviving animals by free PEG-Lip-TQ after 14 days in phase 1, (**G**) Average body weight by PEG-Lip-TQ during the experiment period in phase 2, (**H**) Average body weight of surviving animals by free PEG-Lip-TQ after 14 days in phase 2, (**I**) LD_50_ of free TQ and PEG-Lip-TQ. The values are expressed as mean ± SE of three independent experiments. * Significant difference between the treated groups, *p*-value < 0.05, **** Significant difference between the treated groups, *p*-value < 0.0001.

The phase 1 in vivo oral toxicity data showed that 100% and 66% mortality were observed by TQ 100 and TQ 1000, respectively (Table S2). Interestingly, no mortality was found in PEG-Lip-TQ 100, although a 33% mortality was recorded in PEG-Lip-TQ 1000 (Table S4). The doses PEG-Lip-TQ for phase 2 were increased as 600, 1000, 1600, and 2900 mg/kg bw (Table S5), while it was reduced for free TQ as 20, 40, 100, and 160 mg/kg bw (Table S3). The mortality data from both of the phases suggested the IC_50_ for the free TQ as well as PEG-Lip-TQ as 89.5 and 775 mg/kg b.w, respectively (Tables S2–S5, Figure 8I). Noticeably, a several fold increase (~9) was recorded in the LD_50_, evidently suggesting the use of PEG-Lip-TQ in the treatment strategy with TQ. In the phase study, no significant change in the body weight was observed in TQ-0 as well as TQ-10, while a significant weight reduction (>20%, *p* < 0.05) was recorded in TQ-100 (Figure 8A,B). Remarkably, no significant decrease change in the body weight was observed in the animals exposed to varying doses of PEG-Lip-TQ in phase 1 (Figure 8E,F). The phase 2 data showed a significant decrease in the body weight in the animals exposed to TQ 100 and TQ 160, while no significant change in the body weight was observed in TQ 20, TQ 40, as well as TQ 80 (Figure 8C,D). However, no change in the body weight was observed in the surviving animals as 100% was recorded in PEG-Lip-TQ 1600 and PEG-Lip-TQ 2900 animals at 6 and 8 days, respectively, after the start of phase 2 study (Figure 8C,D).

##### Effect of TQ on Leukocytes by Hematological Studies

**Figure 9:**
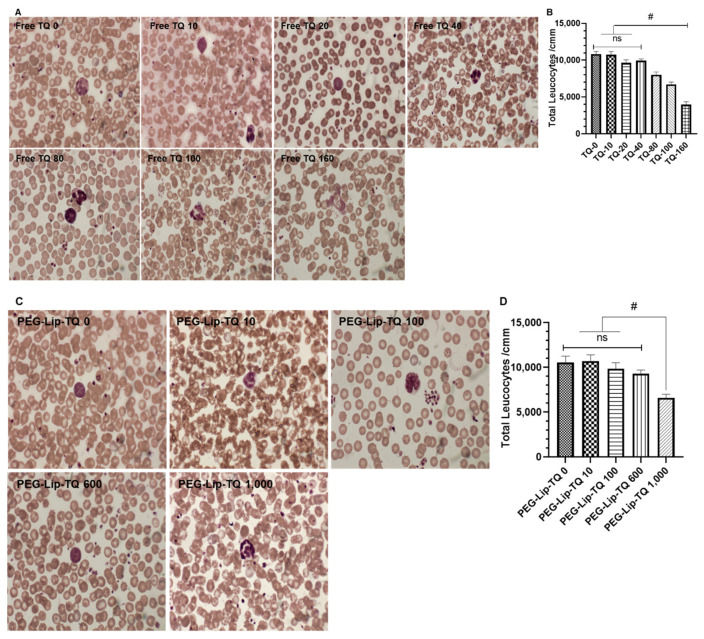
Effect of TQ on lymphocytes structure and total leukocytes count (TLC) of all the surviving animals from phase 1 and phase 2 (**A**) Lymphocytes structure by free TQ, (**B**) TLC by free TQ, (**C**) Lymphocytes structure by PEG-Lip-TQ, (**D**) TLC in by PEG-Lip-TQ. The values are expressed as mean ± SE of three independent experiments. ^NS^ No significance within the groups, ^#^ Significant difference between the treated groups.

The Leishman staining of the leukocytes in all surviving animals of both phases showed ruptured and degranulation of the cells in the animals treated with TQ-80, TQ, 100, and TQ 160, while vacuolization could also be seen in TQ 160 (Figure 9A). The hematological data revealed a significant decrease in the total leucocytes count in the animals exposed to TQ-80, TQ-100, and TQ-160 in comparison to TQ-0 (Figure 9B). Noticeably, no structural change was observed in the cells exposed to PEG-Lip-TQ doses except PEG-Lip-TQ 1000 as ruptured cells could be seen in it (Figure 9C). The hematological data of total leukocytes also showed the same pattern as a significant reduction was measured only in the animals exposed to PEG-Lip-TQ 1000 (Figure 9D).

##### Effect of TQ on Relative Organ Weight

**Figure 10:**
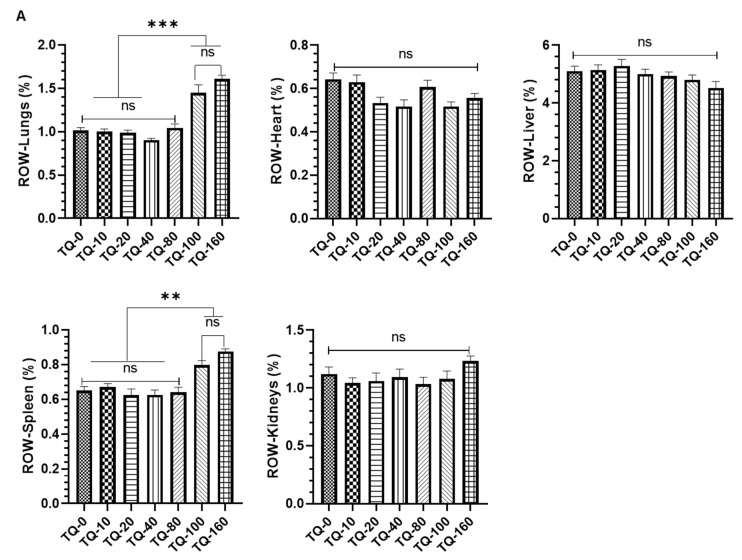

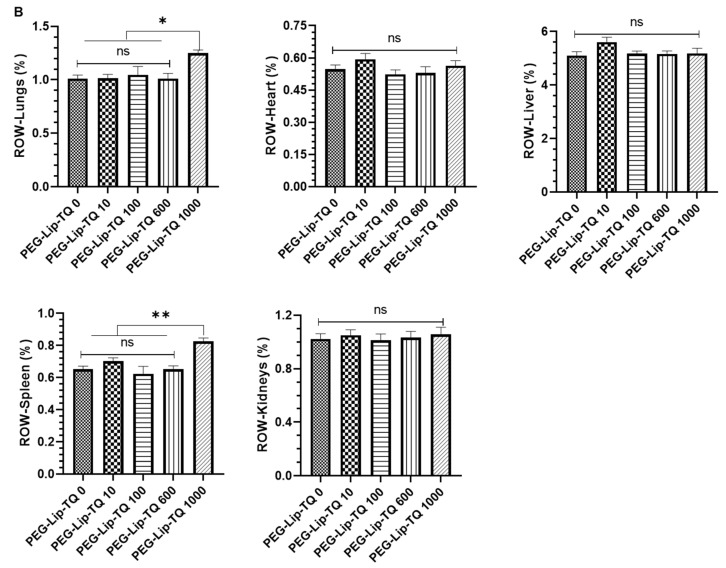
Effect of TQ on the relative organ weight of vital organs (lungs, heart, liver, spleen, and kidneys) in all the surviving animals from phase 1 and phase 2. The quantitative analysis of ROW from the animals exposed to (**A**) free TQ (**B**) PEG-Lip-TQ. The values are expressed as mean ± SE of three independent experiments. ^NS^ No significance within the groups, * Significant difference between the treated groups, *p*-value < 0.05, ** Significant difference between the treated groups, *p*-value < 0.001, *** Significant difference between the treated groups, *p*-value < 0.0001.

The relative organ weight (ROW) showed significant changes in the lungs and the spleen of the animals exposed to TQ-100 and TQ-160. However, no significant change was observed in the ROW of the heart, liver, and kidneys in any of the treated group of free TQs. As depicted in Figure 10A, a 43% and 58% increase in the ROW were calculated in the lungs of TQ-100 and TQ-160, respectively, clearly indicating the toxicity of TQ in the lungs at these concentrations. The ROW of spleen data showed a 22.5% in TQ-100 and ~35% increase in TQ-160 (Figure 10A), which directed us to make the formulations of TQ to avoid such a type of toxicity, while using it is frequently used in the ailment of several diseases including lung cancer. The ROW results of the surviving animals exposed to PEG-Lip-TQ revealed a 23.76% and 26.5% increase in the ROW of lungs and spleen, respectively, by PEG-Lip-TQ 100, while no change was observed in any of the other doses in the ROW (Figure 10B).

## 4. Discussion

The present study is the first to develop the TQ-containing long-circulating PEGylated stealth liposomes, investigating the entrapment efficiency, stability, and release kinetics in the serum. The hydrophobic nature of TQ showed high entrapment efficiency of 96%. The rigidity of lipid bilayers makes the liposomes stable, depending on the choice of lipids and its molar concentration with a high phase transition temperature (Tc) [46,47,48,49]. The presence of cholesterol in a suitable amount with phospholipids is the important factor in the stability of liposomes. However, it has not been defined, but several studies suggested a 2:1 ratio of phospholipid and cholesterol to make the liposomes most stable and efficient [50,51,52]. Moreover, recently, we reported the high stability of liposomes in 49:21 molar ratios of DSPC and cholesterol [37]. Irrespective of the high stability of liposomes, the minimal uptake of liposomes by RES is a major challenge in the preparation of novel formulations, while targeting extra RES tissues is the targeting of extra RES tissues. The coating of liposomes with PEGylated lipids makes sterically stabilized liposomes, enabling them to remain in the circulation for a longer period and release the drug to the tumor cells [53,54,55]. The cumulative release data of PEGylated liposomes showed only 3.5% and 15% TQ in the PBS and serum, respectively, at 37 °C, clearly indicating the high stability and long circulation of the formulation (Figure 5A,B). The release of only a small amount might be associated with the high lipophilicity of TQ as it strongly attached to the lipids. Earlier, some studies also showed a low concentration of TQ release due to the hydrophobic nature of the compound [20,56].

The cytotoxic effect of PEG-Lip-TQ showed a tremendous decrease in the IC_50_ of TQ, while comparing it with free TQ. The cell viability assay also revealed the great efficacy of PEG-Lip-TQ with time as more than 40% and 50% lesser doses were required for the IC_50_, while treating the A549 and H460 lung cancer cells for 72 h, in comparison to 48 h. The persistent bioavailability of the TQ in PEGylated liposomes might have increased the cytotoxicity of PEG-Lip-TQ (Figure 6). Noticeably, no study has yet reported the effect of TQ-loaded liposomes against any cancer cells in a time-dependent manner.

According to OECD, the in vivo oral acute toxicity assay is one of the significant criteria for the primary screening of the drug to determine the LD_50_ and safety level of doses before making the treatment strategy [32,57,58,59]. In the analysis using ProTox-II, the toxicity of TQ was found to be inactive in all the tested toxicities. However, the induction of mitochondrial membrane potential (MMP) was observed in the analysis, confirming its toxicity through the MMP-mediated pathway, which may lead to apoptosis and/or necrosis of the cells. Moreover, the variations in the LD_50_ were noticed by ProTox-II as well as three methods of the T.E.S.T program. The ProTox-II projected 2400 mg/kg, while T.E.S.T predicted 816.9, 1004.5, and 664.4 mg/kg by the hierarchical clustering, consensus, and nearest neighbour methods, respectively (Table 3). The LD_50_ projected by the nearest T.E.S.T program clearly indicated a toxicity of TQ of even less than 1000 mg/kg (Table 3). The results from the in vivo acute oral toxicity assay in Swiss albino mice demonstrated that the LD_50_ of free TQ and PEG-Lip-TQ was 89.5 mg/kg and 775 mg/kg b.w, respectively. The toxic effect of free TQ was also observed in ROW of the lungs and spleen in the animals exposed to TQ-100 and TQ-160 but no toxicity was noticed in PEG-Lip-TQ lesser than PEG-Lip-TQ 1000 (Figure 10). As per the classifications of the drugs suggested by OECD, the TQ will be shifted from class 3 to class 4, when entrapped in long-circulating PEGylated liposomes. Remarkably, PEG-Lip-TQ demonstrated a 7-fold increase in the maximum tolerated dose while comparing free TQ (Tables S2–S5). Earlier, Badry et al. 1998, reported the LD_50_ of TQ as 2400 mg/kg, but our survival data revealed 100% mortality in the animals exposed to the dose of TQ 1000 (Table 3) [60]. Besides, the survival rate was found to be 0% in the animals treated with more than 1000 mg/kg by PEG-Lip-TQ (Table S5).

A molecular docking investigation was carried against many pharmacological targets linked to anticancer drug development to acquire an insight into the predictive basis for the probable interaction mechanisms of TQ. To our knowledge, this is the first research to use the AutoDock 4.2 molecular docking technology to characterize the molecular interactions profile of this molecule against multiple drug targets relevant to carcinogenesis. TQ exhibited the highest affinity for the Hsp90 protein, which had a binding energy of −6.05 kcal/mol, and the lowest affinity for the caspase-3 enzyme, demonstrating a binding energy of −1.19 kcal/mol. Hsp90 plays an essential role in oncogenic signaling, making it an intriguing target for cancer treatment. Therefore, inhibition of Hsp90 activity concurrently disrupts various signal transduction pathways that are critical for tumor growth and survival [61]. Molecular docking is a robust computational technique for inferring the intermolecular interactions between ligands and proteins, but because the solvation penalty associated with the binding cannot be accurately assessed [62], further research is needed to justify the approximation of the binding energy. Superimposition of the docked TQ and native co-crystallized inhibitor of the Hsp90 protein, 5-(3,4-dichloro-phenoxy)-benzene-1,3-diol, exhibited a root-mean square deviation of 3.23 Å (Figure 4), which further confirms the potential of TQ-based ligand to be developed as an Hsp90 inhibitor. Research into the TQ’s particular anticancer mode of action as well as lead optimization might be sparked by the current findings.

The present study demonstrated the efficacy of PEG-Lip-TQ in terms of bioavailabilty, cytotoxic activities against lung cancer cells, and decreased the toxicity as well. Moreover, the molecular docking data suggested several molecular targets of TQ that should be evaluated in the lung cancer system in vitro as well as in vivo.

## 5. Conclusions

The results of this study indicate that the pharmacological and efficacy profile of PEG-lip-TQ is superior to free TQ as demonstrated by release kinetics, in vitro lung cancer cytotoxicity, and in vivo safety level of doses as well. Further studies are required to understand the molecular mechanism of PEG-Lip-TQ in the treatment of lung cancer in vitro as well as in vivo, focusing on the molecular targets of TQ, as suggested by molecular docking data. This study will also pave the way to explore the effect of TQ formulation in the treatment of lung cancer in clinical settings. The synergistic effect of these TQ-loaded liposomes should also be evaluated in combination with least toxic concentration of various chemotherapeutic drugs.

## Figures and Tables

**Table 1:** Free TQ and PEG-Lip-TQ doses in both phases of the in vivo oral acute toxicity assay in Swiss albino mice.

Phases	Free TQ (mg/kg/b.w)	PEG-Lip-TQ (mg/kg/b.w)
Phase-1	0, 10, 100, 1000	0, 10, 100, 1000
Phase-2	0, 20, 40, 80, 100, 160	0, 600, 1000, 1600, 2900

**Table 2:** TQ toxicity determined by ProTox-II webserver.

S.N	Classification	Target	Prediction	Probability
1.	Organ toxicity	Hepatotoxicity	Inactive	0.63
2.	Toxicity end points	Carcinogenicity	Inactive	0.63
		Immunotoxicity	Inactive	0.97
		Mutagenicity	Inactive	0.91
		Cytotoxicity	Inactive	0.78
3.	Nuclear receptor signaling pathways	Aryl hydrocarbon receptor	Inactive	0.93
		Androgen receptor	Inactive	0.99
		Androgen receptor ligand binding domain	Inactive	0.99
		Aromatase	Inactive	0.99
		Estrogen receptor alpha	Inactive	0.96
		Estrogen receptor ligand binding domain	Inactive	0.93
		Peroxisome proliferator activated receptor gamma	Inactive	0.99
4.	Stress response pathways	Nuclear factor (erythroid-derived 2)-like 2/antioxidant responsive element (nrf2/ARE)	Inactive	0.93
		Heat shock factor response element (HSE)	Inactive	0.93
		Mitochondrial membrane potential	Active	0.58
		Phosphoprotein (tumor suppressor) p53	Inactive	0.92
		ATPase family AAA domain-containing protein 5 (ATAD5)	Inactive	0.99

**Table 3:** The expected toxicities linked with the TQ as predicted by the T.E.S.T. software (US Environmental Protection Agency).

Toxicity Parameters		Toxicity Results
Consensus method	Oral rat LD_50_ mg/kg	816.9
	Mutagenicity value	0.19
	Mutagenicity result	Negative
Hierarchical clustering method	Oral rat LD_50_ mg/kg	1004.5
	Mutagenicity value	0.05
	Mutagenicity result	Negative
Nearest neighbor method	Oral rat LD_50_ mg/kg	664.4
	Mutagenicity value	0.33
	Mutagenicity result	Negative

## Data Availability

Not applicable.

## Supplementary Materials

The following supporting information can be downloaded at: https://www.mdpi.com/article/10.3390/pharmaceutics14010153/s1, Table S1: AutoDock 4.2 results of TQ with several anticancer drug targets; Table S2: Effect of free TQ on general appearance and behavioral observations in Phase-1 oral acute toxicity assay in Swiss albino mice; Table S3: Effect of free TQ on general appearance and behavioural observations in Phase-2 oral acute toxicity assay in Swiss albino mice; Table S4: Effect of PEG-Lip-TQ on general appearance and behavioural observations in Phase-1 oral acute toxicity assay in Swiss albino mice; Table S5: Effect of PEG-Lip-TQ on general appearance and behavioural observations in Phase-2 oral acute toxicity assay in Swiss albino mice; Figure S1. Effect of TQ on relative organ weight (ROW) of vital organs (lungs, heart, liver, spleen and kidneys) in all the surviving animals from phase-1 and phase-2. The representing images of vital organs from the animals exposed to (A) Free TQ (B) PEG-Lip-TQ.

## References

[1] Sung H., Ferlay J., Siegel R.L., Laversanne M., Soerjomataram I., Jemal A., Bray F. (2021). Global Cancer Statistics 2020: GLOBOCAN Estimates of Incidence and Mortality Worldwide for 36 Cancers in 185 Countries. CA Cancer J. Clin..

[2] Pucci C., Martinelli C., Ciofani G. (2019). Innovative approaches for cancer treatment: Current perspectives and new challenges. Ecancermedicalscience.

[3] Hameed R., Khan A., Khan S., Perveen S. (2019). Computational Approaches Towards Kinases as Attractive Targets for Anticancer Drug Discovery and Development. Anti Cancer Agents Med. Chem..

[4] Thomford N.E., Senthebane D.A., Rowe A., Munro D., Seele P., Maroyi A., Dzobo K. (2018). Natural Products for Drug Discovery in the 21st Century: Innovations for Novel Drug Discovery. Int. J. Mol. Sci..

[5] Chopra B., Dhingra A.K. (2021). Natural products: A lead for drug discovery and development. Phytother. Res..

[6] Newman D.J., Cragg G.M. (2020). Natural products as sources of new drugs over the nearly four decades from 01/1981 to 09/2019. J. Nat. Prod..

[7] Zhang L., Song J., Kong L., Yuan T., Li W., Zhang W., Hou B., Lu Y., Du G. (2020). The strategies and techniques of drug discovery from natural products. Pharmacol. Ther..

[8] Ma L., Zhang M., Zhao R., Wang D., Ma Y., Li A. (2021). Plant Natural Products: Promising Resources for Cancer Chemoprevention. Molecules.

[9] Block G., Patterson B., Subar A. (1992). Fruit, vegetables, and cancer prevention: A review of the epidemiological evidence. Nutr. Cancer.

[10] Malik S., Singh A., Negi P., Kapoor V.K. (2021). Thymoquinone: A small molecule from nature with high therapeutic potential. Drug Discov. Today.

[11] Gali-Muhtasib H., Roessner A., Schneider-Stock R. (2006). Thymoquinone: A promising anti-cancer drug from natural sources. Int. J. Biochem. Cell Biol..

[12] Khan M.A., Younus H. (2019). Thymoquinone Shows the Diverse Therapeutic Actions by Modulating Multiple Cell Signaling Pathways: Single Drug for Multiple Targets. Curr. Pharm. Biotechnol..

[13] Khader M., Eckl P.M. (2014). Thymoquinone: An emerging natural drug with a wide range of medical applications. Iran. J. Basic Med. Sci..

[14] Botnick I., Xue W., Bar E., Ibdah M., Schwartz A., Joel D.M., Lev E., Fait A., Lewinsohn E. (2012). Distribution of Primary and Specialized Metabolites in Nigella sativa Seeds, a Spice with Vast Traditional and Historical Uses. Molecules.

[15] Ansary J., Giampieri F., Forbes-Hernandez T.Y., Regolo L., Quinzi D., Villar S.G., Villena E.G., Pifarre K.T., Alvarez-Suarez J.M., Battino M. (2021). Nutritional Value and Preventive Role of *Nigella sativa* L. and Its Main Component Thymoquinone in Cancer: An Evidenced-Based Review of Preclinical and Clinical Studies. Molecules.

[16] Khan A., Tania M., Fu S., Fu J. (2017). Thymoquinone, as an anticancer molecule: From basic research to clinical investigation. Oncotarget.

[17] Rathore C., Rathbone M.J., Chellappan D.K., Tambuwala M.M., Pinto T.D.J.A., Dureja H., Hemrajani C., Gupta G., Dua K., Negi P. (2020). Nanocarriers: More than tour de force for thymoquinone. Expert Opin. Drug Deliv..

[18] Salmani J.M.M., Asghar S., Lv H., Zhou J. (2014). Aqueous Solubility and Degradation Kinetics of the Phytochemical Anticancer Thymoquinone; Probing the Effects of Solvents, pH and Light. Molecules.

[19] Allemailem K., Alnuqaydan A., Almatroudi A., Alrumaihi F., Aljaghwani A., Khalilullah H., Younus H., Khan A., Khan M. (2021). Safety and Therapeutic Efficacy of Thymoquinone-Loaded Liposomes against Drug-Sensitive and Drug-Resistant *Acinetobacter baumannii*. Pharmaceutics.

[20] Alam Khan M., Aljarbou A.N., Khan A., Younus H. (2015). Liposomal thymoquinone effectively combats fluconazole-resistant Candida albicans in a murine model. Int. J. Biol. Macromol..

[21] Odeh F., Ismail S.I., Abu-Dahab R., Mahmoud I., Al Bawab A. (2012). Thymoquinone in liposomes: A study of loading efficiency and biological activity towards breast cancer. Drug Deliv..

[22] Odeh F., Naffa R., Azzam H., Mahmoud I., Alshaer W., Al Bawab A., Ismail S. (2019). Co-encapsulation of thymoquinone with docetaxel enhances the encapsulation efficiency into PEGylated liposomes and the chemosensitivity of MCF7 breast cancer cells to docetaxel. Heliyon.

[23] Shahraki N., Mehrabian A., Amiri-Darban S., Moosavian S.A., Jaafari M.R. (2021). Preparation and characterization of PEGylated liposomal Doxorubicin targeted with leptin-derived peptide and evaluation of their anti-tumor effects, in vitro and in vivo in mice bearing C26 colon carcinoma. Colloids Surfaces B Biointerfaces.

[24] Pozzi D., Colapicchioni V., Caracciolo G., Piovesana S., Capriotti A.L., Palchetti S., De Grossi S., Riccioli A., Amenitsch H., Laganà A. (2014). Effect of polyethyleneglycol (PEG) chain length on the bio–nano-interactions between PEGylated lipid nanoparticles and biological fluids: From nanostructure to uptake in cancer cells. Nanoscale.

[25] Chow T.-H., Lin Y.-Y., Hwang J.-J., Wang H.-E., Tseng Y.-L., Wang S.-J., Liu R.S., Lin W.J., Yang C.S., Ting G. (2009). Improvement of biodistribution and therapeutic index via increase of polyethylene glycol on drug-carrying liposomes in an HT-29/luc xenografted mouse model. Anticancer. Res..

[26] Fatima N., Nayeem N. (2016). Toxic Effects as a Result of Herbal Medicine Intake. Toxicology—New Aspects to This Scientific Conundrum.

[27] Zhang J., Onakpoya I.J., Posadzki P., Eddouks M. (2015). The Safety of Herbal Medicine: From Prejudice to Evidence. Evid. Based Complement. Altern. Med..

[28] Ekor M. (2014). The growing use of herbal medicines: Issues relating to adverse reactions and challenges in monitoring safety. Front. Pharmacol..

[29] Rousseaux C.G., Schachter H. (2003). Regulatory issues concerning the safety, efficacy and quality of herbal remedies. Birth Defects Res. Part B Dev. Reprod. Toxicol..

[30] (2018). OECD Test No. 452: Chronic Toxicity Studies.

[31] Lorke D. (1983). A new approach to practical acute toxicity testing. Arch. Toxicol..

[32] (2008). OECD Test No. 425: Acute Oral Toxicity: Up-and-Down Procedure.

[33] Tang J., Guo F., Ding Y. (2020). The Computational Models of Drug-target Interaction Prediction. Protein Pept. Lett..

[34] Ferrero E., Dunham I., Sanseau P. (2017). In silico prediction of novel therapeutic targets using gene–disease association data. J. Transl. Med..

[35] Tanzifi M., Yaraki M.T., Beiramzadeh Z., Saremi L.H., Najafifard M., Moradi H., Mansouri M., Karami M., Bazgir H. (2020). Carboxymethyl cellulose improved adsorption capacity of polypyrrole/CMC composite nanoparticles for removal of reactive dyes: Experimental optimization and DFT calculation. Chemosphere.

[36] Azam F., Abodabos H.S., Taban I.M., Rfieda A.R., Mahmood D., Anwar J., Khan S., Sizochenko N., Poli G., Tuccinardi T. (2019). Rutin as promising drug for the treatment of Parkinson’s disease: An assessment of MAO-B inhibitory potential by docking, molecular dynamics and DFT studies. Mol. Simul..

[37] Khan A., Aljarbou A.N., Aldebasi Y.H., Allemeilam K.S., A Alsahly M., Khan S., Alruwetei A.M., A Khan M. (2020). Fatty Acid Synthase (FASN) siRNA-Encapsulated-Her-2 Targeted Fab’-Immunoliposomes for Gene Silencing in Breast Cancer Cells. Int. J. Nanomed..

[38] Morris G.M., Goodsell D.S., Halliday R.S., Huey R., Hart W.E., Belew R.K., Olson A.J. (1998). Automated docking using a Lamarckian genetic algorithm and an empirical binding free energy function. J. Comput. Chem..

[39] Azam F. (2021). Elucidation of Teicoplanin Interactions with Drug Targets Related to COVID-19. Antibiotics.

[40] Fahmy N.M., Al-Sayed E., Moghannem S., Azam F., El-Shazly M., Singab A.N. (2020). Breaking Down the Barriers to a Natural Antiviral Agent: Antiviral Activity and Molecular Docking of Erythrina speciosa Extract, Fractions, and the Major Compound. Chem. Biodivers..

[41] Khan M.A., Aldebasi Y.H., Alsuhaibani S.A., Alsahli M.A., Alzohairy M.A., Khan A., Younus H. (2018). Therapeutic potential of thymoquinone liposomes against the systemic infection of Candida albicans in diabetic mice. PLoS ONE.

[42] Banerjee P., O Eckert A., Schrey A.K., Preissner R. (2018). ProTox-II: A webserver for the prediction of toxicity of chemicals. Nucleic Acids Res..

[43] Martin T. (2016). Toxicity Estimation Software Tool (TEST). https://www.epa.gov/chemical-research/toxicity-estimation-software-tool-test.

[44] Hussain S., Azam F., Eldarrat H.A., Alkskas I., Mayoof J.A., Dammona J.M., Ismail H., Ali M., Arif M., Haque A. (2020). Anti-inflammatory, analgesic and molecular docking studies of Lanostanoic acid 3-O-α-D-glycopyranoside isolated from Helichrysum stoechas. Arab. J. Chem..

[45] Jeon J., Kang S., Kim H.U. (2021). Predicting biochemical and physiological effects of natural products from molecular structures using machine learning. Nat. Prod. Rep..

[46] Li M., Du C., Guo N., Teng Y., Meng X., Sun H., Li S., Yu P., Galons H. (2019). Composition design and medical application of liposomes. Eur. J. Med. Chem..

[47] Ahmed K.S., Hussein S.A., Ali A., Korma S.A., Lipeng Q., Jinghua C. (2018). Liposome: Composition, characterisation, preparation, and recent innovation in clinical applications. J. Drug Target..

[48] Anderson M., Omri A. (2004). The Effect of Different Lipid Components on the In Vitro Stability and Release Kinetics of Liposome Formulations. Drug Deliv..

[49] Crommelin D.J. (1984). Influence of Lipid Composition and Ionic Strength on the Physical Stability of Liposomes. J. Pharm. Sci..

[50] Briuglia M.-L., Rotella C.M., McFarlane A., Lamprou D.A. (2015). Influence of cholesterol on liposome stability and on in vitro drug release. Drug Deliv. Transl. Res..

[51] Magarkar A., Dhawan V., Kallinteri P., Viitala T., Elmowafy M., Róg T., Bunker A. (2014). Cholesterol level affects surface charge of lipid membranes in saline solution. Sci. Rep..

[52] Papahadjopoulos D., Cowden M., Kimelberg H. (1973). Role of cholesterol in membranes effects on phospholipid-protein interactions, membrane permeability and enzymatic activity. Biochim. Biophys. Acta (BBA) Biomembr..

[53] Vargason A.M., Anselmo A.C., Mitragotri S. (2021). The evolution of commercial drug delivery technologies. Nat. Biomed. Eng..

[54] Singh A., Neupane Y.R., Shafi S., Mangla B., Kohli K. (2020). PEGylated liposomes as an emerging therapeutic platform for oral nanomedicine in cancer therapy: In vitro and in vivo assessment. J. Mol. Liq..

[55] Jiménez-López J., Bravo-Caparrós I., Cabeza L., Nieto F.R., Ortiz R., Perazzoli G., Fernández-Segura E., Cañizares F.J., Baeyens J.M., Melguizo C. (2021). Paclitaxel antitumor effect improvement in lung cancer and prevention of the painful neuropathy using large pegylated cationic liposomes. Biomed. Pharmacother..

[56] Mostafa M., Alaaeldin E., Aly U.F., Sarhan H.A. (2018). Optimization and Characterization of Thymoquinone-Loaded Liposomes with Enhanced Topical Anti-inflammatory Activity. AAPS PharmSciTech.

[57] Hernández-Alvarado R.B., Madariaga-Mazón A., Martinez-Mayorga K. (2019). Prediction of toxicity of secondary metabolites. Phys. Sci. Rev..

[58] Madariaga-Mazón A., Hernández-Alvarado R.B., Noriega-Colima K.O., Osnaya-Hernández A., Martinez-Mayorga K. (2019). Toxicity of secondary metabolites. Phys. Sci. Rev..

[59] Raies A., Bajic V.B. (2016). In silicotoxicology: Computational methods for the prediction of chemical toxicity. WIREs Comput. Mol. Sci..

[60] Badary O., Al-Shabanah O.A., Nagi M.N., Al-Bekairi A.M., Elmazar M.M. (1998). Acute and subchronic toxicity of thymoquinone in mice. Drug Dev. Res..

[61] Moser C., Lang S.A., Stoeltzing O. (2009). Heat-shock protein 90 (Hsp90) as a molecular target for therapy of gastrointestinal cancer. Anticancer Res..

[62] Pantsar T., Poso A. (2018). Binding Affinity via Docking: Fact and Fiction. Molecules.

