# Supplementary material for: Safety, Stability, and Therapeutic Efficacy of Long-Circulating TQ-Incorporated Liposomes: Implication in the Treatment of Lung Cancer
Source: Pharmaceutics. 2022 Jan 9;14(1):153. doi: 10.3390/pharmaceutics14010153 (PMC8778344; doi:10.3390/pharmaceutics14010153)
Supplement: Supplementary file 1 [file pharmaceutics-14-00153-s001.zip › pharmaceutics-1507049-supplementary.pdf]

pharmaceutics

Supplementary Materials: Safety, Stability and Therapeutic Efficacy of Long-Circulating TQ-Incorporated Liposomes: Implication in the Treatment of Lung Cancer

Arif Khan, Mohammed A. Alsahli, Mohammad A. Aljasir, Hamzah Maswadeh, Mugahid A. Mobark, Faizul Azam, Khaled S. Allemailem, Faris Alrumaihi, Fahad A. Alhumaydhi, Ameen S. S. Alwashmi, Ahmed A. Almatroudi, Mahdi H. Alsugoor and Masood A. Khan

Table S1. AutoDock 4.2 results of TQ with several anticancer drug targets.

S.N.	Targets	PDB Code	Binding Energy of TQ in kcal/mol
1	Matrix Metalloproteinase-2 (MMP-2)	1HOV	-5.34
2	Chk1	2R0U	-3.05
3	Cyclin A	6GUE	-5.61
4	Apoptosis regulator Bcl-2	4LXD	-4.64
5	Src kinase	2BDF	-2.66
6	phosphatidylinositol-3 kinase alpha	3ZIM	-5.82
7	Heat Shock Protein 90 (Hsp90)	4BQG	-6.05
8	JAK2	3KCK	-4.28
9	Caspase 3	1RE1	-1.19
10	EGFR	1M17	-1.99
11	GTPase HRas	6BVM	-3.77
12	VEGFR2 kinase	4AG8	-5.41

Table S2. Effect of free TQ on general appearance and behavioral observations in Phase-1 oral acute toxicity assay in Swiss albino mice.

	TQ 0	TQ 10	TQ 100	TQ 1000
Skin and fur	NO	NO	PIL	PIL
Eyes	NO	NO	BOE	BOE
Mucous membrane	NO	NO	NO	NO
Behavioral patterns	NO	NO	Ab	Ab
Salivation	NO	NO	NO	NO
Lethargy/Sleep	NO	NO	Ab	Ab
Diarrhea	NO	NO	NO	NO
Mortality	0/3	0/3	2/3	3/3

Abbreviations: Not observed (NO); Abnormality detected (Ab); Piloerection (PIL); Blinking of eyes (BOE)

Table S3. Effect of free TQ on general appearance and behavioural observations in Phase-2 oral acute toxicity assay in Swiss albino mice.

Observations	TQ 0	TQ 20	TQ 40	TQ 80	TQ 100	TQ 160
Skin and fur	NO	NO	NO	NO	PIL	PIL
Eyes	NO	NO	NO	BOE	BOE	BOE
Mucous membrane	NO	NO	NO	NO	NO	NO
Behavioral patterns	NO	NO	NO	NO	Ab	Ab
Salivation	NO	NO	NO	NO	NO	NO
Lethargy/Sleep	NO	NO	NO	Ab	Ab	Ab

Diarrhea	NO	NO	NO	NO	NO	NO
Mortality	0/3	0/3	0/3	0/3	2/3	2/3

Table S4. Effect of PEG-Lip-TQ on general appearance and behavioural observations in Phase-1 oral acute toxicity assay in Swiss albino mice.

Observations	PEG-Lip-TQ 0	PEG-Lip-TQ 10	PEG-Lip-TQ 100	PEG-Lip-TQ 1000
Skin and fur	NO	NO	NO	PIL
Eyes	NO	NO	NO	BOE
Mucous membrane	NO	NO	NO	NO
Behavioral patterns	NO	NO	NO	Ab
Salivation	NO	NO	NO	NO
Lethargy/Sleep	NO	NO	NO	Ab
Diarrhea	NO	NO	NO	NO
Mortality	0/3	0/3	0/3	1/3

Table S5. Effect of PEG-Lip-TQ on general appearance and behavioural observations in Phase-2 oral acute toxicity assay in Swiss albino mice.

Observations	PEG-Lip-TQ 0	PEG-Lip-TQ 600	PEG-Lip-TQ 1000	PEG-Lip-TQ 1600	PEG-Lip-TQ 2900
Skin and fur	NO	PIL	PIL	PIL	NO
Eyes	NO	BOE	BOE	BOE	BOE
Mucous membrane	NO	NO	NO	NO	NO
Behavioral patterns	NO	NO	Ab	Ab	Ab
Salivation	NO	NO	NO	NO	NO
Lethargy/Sleep	NO	NO	Ab	Ab	Ab
Diarrhea	NO	NO	NO	NO	NO
Mortality	0/3	0/3	0/3	3/3	3/3

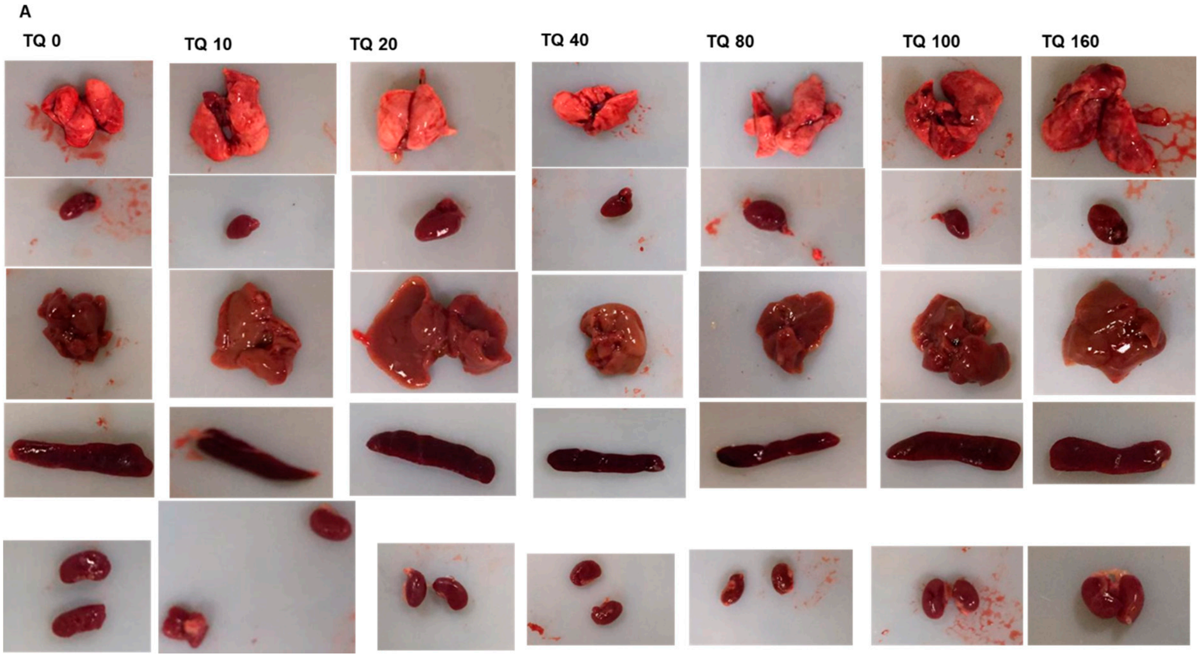

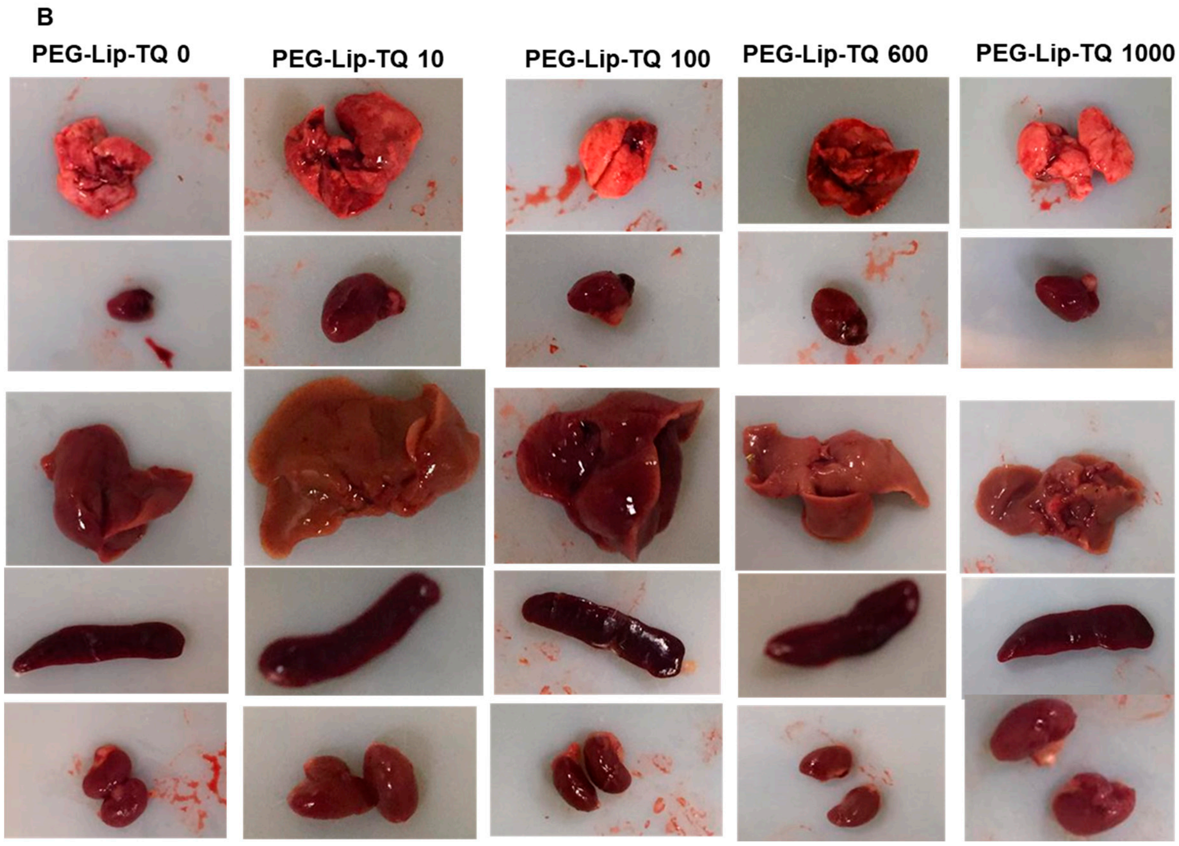

Figure S1. Effect of TQ on relative organ weight (ROW) of vital organs (lungs, heart, liver, spleen and kidneys) in all the surviving animals from phase-1 and phase-2. The representing images of vital organs from the animals exposed to **(A)** Free TQ **(B)** PEG-Lip-TQ.
